# Revolution in Cell Therapy: In Vivo Chimeric-Antigen-Receptor-T-Cell Therapy Breakthroughs and Promises for the Future

**DOI:** 10.34133/research.0917

**Published:** 2025-10-09

**Authors:** Mingyu Lai, Wenxia Shao, Jianhua Mao, Qing Ye

**Affiliations:** ^1^ Department of Nephrology, Children’s Hospital, and Liangzhu Laboratory, Zhejiang University School of Medicine, Hangzhou 310052, China.; ^2^ The First Clinical Medicine College of Lanzhou University, Lanzhou 730030, China.; ^3^ Affiliated Hangzhou First People’s Hospital, Westlake University School of Medicine, Hangzhou 310006, China.; ^4^ Department of Nephrology, The First Affiliated Hospital of Zhejiang Chinese Medical University (Zhejiang Provincial Hospital of Traditional Chinese Medicine), Hangzhou, Zhejiang 310003, China.; ^5^ Department of Laboratory Medicine, Children’s Hospital, and Liangzhu Laboratory, Zhejiang University School of Medicine, Hangzhou 310052, China.

## Abstract

Chimeric-antigen-receptor (CAR)-T-cell therapy has achieved important results in the treatment of hematological tumors, but traditional CAR-T-cell therapy has the problems of complicated in vitro preparation processes, high cost, low T-cell function in patients, difficulty in multiple dosing, and limited treatment efficacy in solid tumors. In vivo CAR-T-cell therapy has emerged as needed. The CAR gene component is directly delivered to T cells in the host through the delivery system to achieve in situ reprogramming, avoids in vitro manipulation, and has important advantages in terms of the timeliness of treatment, economic feasibility, and persistence of treatment. This paper describes the current state of research on in vivo CAR-T-cell therapy, including the development of delivery systems and the application of CAR-T-cell therapy in treating hematological malignancies, solid tumors, autoimmune diseases, and infectious diseases, as well as discussions on efficient delivery, safety regulation, persistence and functional optimization, and overcoming the tumor microenvironment. It also explores innovative solutions, which hold promise for the future development of in vivo CAR-T-cell therapy, particularly in terms of technological breakthroughs, expansion of treatment indications, and industrialization.

## Introduction

As an innovative immunotherapy method, chimeric-antigen-receptor (CAR)-T-cell therapy has achieved important milestones in the treatment of hematological tumors, especially B-cell malignancies. In this type of therapy, T cells are isolated from patients, modified in vitro via genetic engineering techniques, and then reinfused into patients to achieve specific recognition and killing of cancer cells. This process provides new hope for patients whose traditional treatments are ineffective [1]. However, as CAR-T-cell therapy is more widely applied in clinical practice, its core limitations are becoming more apparent. First, complicated in vitro preparation processes constitute the main technical bottleneck of traditional CAR-T-cell therapy. Good manufacturing practice (GMP) standards must be strictly followed throughout the entire process, from T-cell collection, activation, and genetic modification to expansion and return infusion, which is time-consuming and expensive. This process usually takes several weeks; patients with rapidly progressive disease may not be able to receive treatment in time [2]. High production costs have limited the popularity of CAR-T-cell therapy and thus its application in a wider patient population. Second, in some patients, T-cell function may be impaired, leading to insufficient starting materials or a tendency to undergo exhaustion during in vitro expansion, thereby affecting the therapeutic effect [3]. In addition, multiple doses of traditional CAR-T-cell therapy are difficult to achieve because repeated infusions may lead to an immune response or T-cell exhaustion, further limiting its application in long-term treatment. Although CAR-T-cell therapy has shown important results in treating hematological tumors, its effectiveness in solid tumors remains limited, primarily due to the immunosuppressive microenvironment and the insufficient penetration of CAR-T cells [4].

**Fig. 1.**
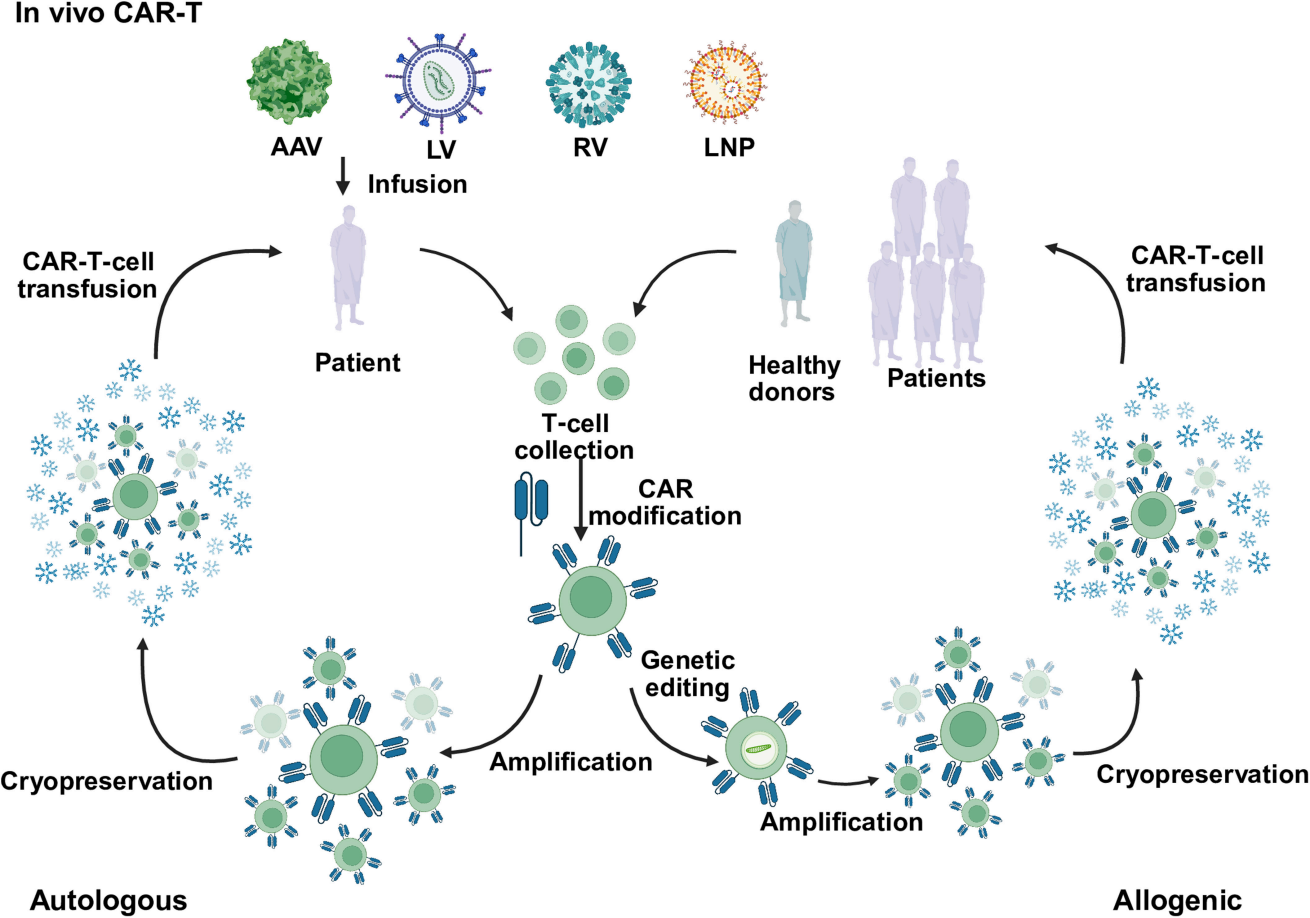
High-level process overview of autologous, allogeneic, and in vivo chimeric-antigen-receptor (CAR)-T therapies. Autologous CAR-T therapy commences with leukapheresis of the patient, followed by in vitro T-cell activation, integration of the CAR gene via viral or nonviral methods, a 2- to 3-week culture expansion period, lymphodepleting chemotherapy, and subsequent reinfusion. Allogeneic CAR-T therapy begins with T cells from a healthy donor undergoing T-cell receptor (TCR) and human leukocyte antigen (HLA) gene editing to reduce the risks of graft-versus-host disease and host rejection. This is followed by the insertion of the CAR construct and large-scale bioreactor expansion. These cells are typically infused without pretreatment, although lymphodepleting chemotherapy may be used in certain contexts to improve engraftment. In vivo CAR-T therapy involves the in situ transduction and activation of T cells within the host, achieved through the intravenous injection of CAR-encoded vectors. AAV, adeno-associated virus; LV, lentivirus; RV, retrovirus; LNP, lipid nanoparticle.

To overcome the limitations of traditional CAR-T-cell therapy, in vivo CAR-T-cell therapy has been developed. The core concept is to use an in vivo delivery system to introduce the CAR gene directly into T cells within the host, enabling in situ reprogramming and avoiding in vitro manipulations. This strategy has brought breakthrough advantages in multiple aspects: in terms of the timeliness of treatment, the CAR-T cells generated in vivo can achieve immediate treatment and considerably shorten the treatment cycle; in terms of economic feasibility, simplifying the process considerably reduces production costs and improves the efficiency of treatment; in terms of the persistence of treatment, the repeatable dosing mechanism can effectively cope with the attenuation of efficacy or relapse after a single treatment; and more importantly, the use of endogenous healthy T cells avoids cell exhaustion caused by in vitro expansion. This offers a novel approach to improving the safety and efficacy of treatment [5]. The research goal of in vivo CAR-T-cell therapy is focused on achieving safe, efficient, and controllable T-cell reprogramming in vivo. By optimizing the delivery system, improving the targeting, and enhancing the persistence and antitumor activity of CAR-T cells, in vivo CAR-T-cell therapy is expected to play an important role in future cancer treatment, especially through breakthroughs in the treatment of solid tumors (Fig. 1).

This paper systematically elaborates the research status and development prospects of in vivo CAR-T-cell therapy; aims to provide some insights into this technology by discussing the core mechanism, disease application, clinical translation potential, and major scientific challenges; and discusses the corresponding cutting-edge solutions. We provide theoretical support and strategic guidance for our breakthrough innovations.

## The Core Engine of CAR-T Cells In Vivo: Delivery Systems

### Evolution of the CAR structure

**Fig. 2.**
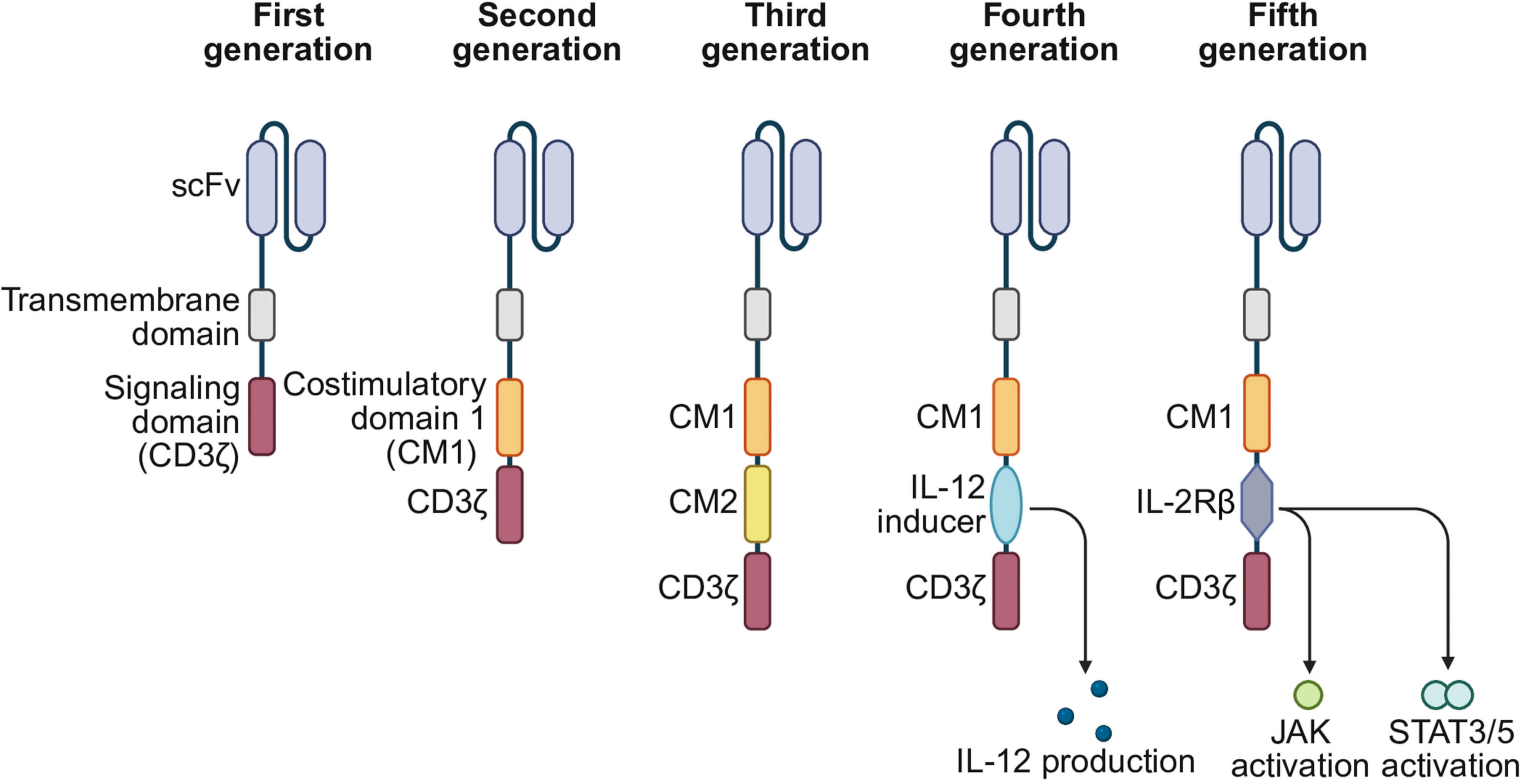
Generations of CAR-T cells. First-generation CARs consist of an single-chain variable fragment (scFv), a transmembrane domain, and a CD3ζ domain, offering basic activation but limited efficacy. Second-generation CARs include a CD28 or 4-1BB costimulatory domain, which considerably enhances T-cell proliferation, persistence, and antitumor activity, particularly against hematologic malignancies. Third-generation designs incorporate dual costimulatory molecules, such as CD28 and 4-1BB, but clinical superiority is not yet clear. Fourth-generation CARs integrate cytokine secretion modules or safety switches to modulate tumor microenvironments. Fifth-generation CARs add cytokine receptor domains that activate Janus kinase–signal transducer and activator of transcription (JAK–STAT) pathways, enhancing T-cell functionality while reducing the risks of cytokine release syndrome, showing promise for solid tumors in ongoing trials. IL-12, interleukin-12‌; STAT3‌/5, signal transducer and activator of transcription 3/5; IL-2Rβ, interleukin-2 receptor beta‌.

Since its inception, CAR-T-cell technology has undergone 5 generations of structural evolution. First-generation CARs consist of an antigen-binding domain (single-chain variable fragment), a transmembrane domain, and a CD3ζ signaling domain, providing only basic T-cell activation signals. However, its therapeutic effect is limited by insufficient cytokine secretion and poor proliferation ability. By introducing the costimulatory molecules CD28 or 4-1BB into first-generation CAR-T cells, second-generation CAR-T cells considerably enhanced the activation, proliferation, and cytokine secretion abilities of T cells while improving their antiapoptotic ability and prolonging cell survival. In particular, it has shown the best efficacy in the treatment of hematological tumors. Third-generation CAR-T cells further combine 2 costimulatory molecules in tandem, such as CD28^+^4-1BB, but their clinical advantage is not important. Building on second-generation CAR-T cells, fourth-generation CAR-T cells have integrated a cytokine secretion module or a safety switch to regulate the microenvironment and improve safety, showing breakthrough potential in the treatment of solid tumors. Based on the second-generation CAR-T cells, fifth-generation CAR-T cells integrate a cytokine receptor signaling domain, such as interleukin-2 receptor beta, which considerably enhances the activity, proliferation, and persistence of T cells by activating the Janus kinase–signal transducer and activator of transcription (JAK–STAT) signaling pathway and optimizes CAR-T characteristics. The release of cytokines reduces the risk of cytokine release syndrome (CRS). This technology has shown important potential in improving the treatment efficacy for solid tumors and is currently in the clinical research stage (Fig. 2) [6].

### Vector system

When CARs are delivered in vivo, a series of stringent criteria must be met, including accurate T-cell targeting, efficient gene editing ability, and low toxicity [7]. Currently, viral vectors, nonviral vectors, and implantable bioscaffolds are the 3 main vector systems that have been widely studied and applied to CAR delivery systems (Table 1) [8].

**Table 1.** Delivery systems of CAR-T cells

Name	Size	Genome	Genome size	Advantages	Limitations
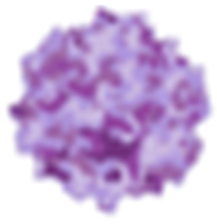 AAV [9–12]	20–25 nm	DNA	~4.7 kb	Low immunogenicity Long-term expression Tissue-targeting serotypes	Small cargo capacity Insertional mutagenesis risk Preexisting antibodies
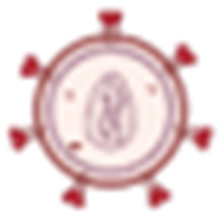 LV [18–20]	80–100 nm	RNA	8–10 kb	High conductivity Large capacity Genomic integration Stable expression	Insertional mutagenesis risk Immunogenic viral proteins High production cost
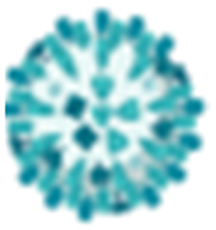 RV [24–26]	80–100 nm	RNA	8–10 kb	Efficient genomic integration Long-term expression	Only transduces dividing cells High insertional risk Immune reactions
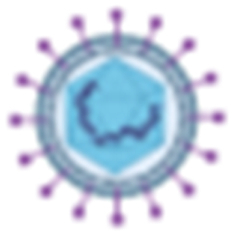 Emerging viral platforms [8,27]	Varies (100–150 nm)	DNA or RNA	Platform dependent	Engineered targeting Immuno-evasion designs Custom promoters	Limited clinical data Unverified long-term safety Scalability challenges
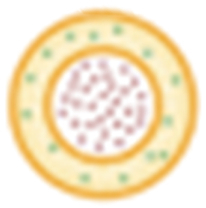 LNPs [29–31]	50–150 nm	Delivery of RNA or pDNA	Varies	No genome integration risk Scalable production Targetable Co-delivery capability	Low T-cell transfection Low drug loading Cold-chain storage required Inflammatory potential
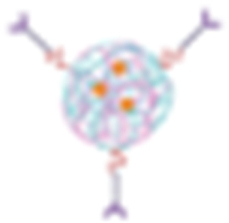 PNPs [34–36]	50–200 nm	Delivery of RNA or pDNA	Varies	Controllable release Surface targetable Nuclease protection	Inefficient release Cytotoxicity risk Storage instability
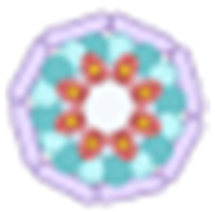 Exosomes [39–41]	30–150 nm	Natural RNA or engineered carriers	Varies	Native biocompatibility CD47-mediated stealth Barrier penetration	Low production yield Poor cargo loading
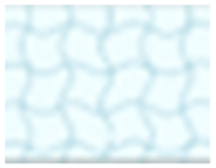 Implantable bioscaffolds [2,43]	Macro size (millimeter–centimeter scale)	ND	ND	Localized/sustained release Pro-survival microenvironment Reduced systemic toxicity	Requires surgery Fibrosis risk Limited cell-loading capacity

pDNA, plasmid DNA; PNPs, polymer nanocarriers; ND, no data

#### Viral vectors

##### Adeno-associated viral vectors

Adeno-associated viral (AAV) vectors are small, non-enveloped viral vectors widely used in gene therapy. They carry 4.7 kb of circular DNA as their genetic material [9]. After AAV vectors enter cells through specific receptors on the cell surface, the therapeutic genes are stably expressed in the episomal form in the nucleus, thereby achieving long-term and continuous gene expression. AAV vectors have important advantages in clinical application. First, they have low immunogenicity and are less likely to trigger attacks of the immune system; second, they can efficiently and durably penetrate multiple organs, including the liver, muscle, and brain [10]. However, the practical application of AAV vectors also faces several key challenges. In terms of safety, although recombinant AAV is designed to remain episomal, there is still a small chance that its genes may integrate into the human DNA strand. This accidental insertion may destroy tumor suppressor genes or activate oncogenes, thereby potentially inducing tumor formation [11,12]. Technical limitations are reflected in the limited capacity of vectors, which limits their ability to deliver complex CAR constructs [13]. In addition, approximately half of the population has natural antibodies in their blood, which can neutralize AAV vectors, limiting their effectiveness [14]. At present, the solutions to these challenges mainly focus on 2 aspects: one is to use the organ navigation ability of different AAV subtypes to achieve precise delivery to specific tissues; the other is to use tissue-specific promoters to activate only in target cells genes to reduce the risk of nonspecific expression [15]. Notably, animal experiments have shown that large doses of AAV may induce hepatocellular carcinoma after it enters the liver [16]. However, there is currently no direct evidence to support the existence of this phenomenon in humans. Therefore, long-term monitoring is needed to assess the relevant risks.

##### Lentiviral vectors

As a “heavy-duty delivery tool” for gene therapy, a lentiviral (LV) vector can overcome the limitations of the cell state and infect cells in the dividing or resting state. Its core function is to permanently integrate therapeutic genes into the DNA of cells to achieve long-term expression. Its greatest advantage is that it has a large-capacity “cargo compartment” of 8 kb, which is enough to load complex CAR structures, and it has the natural ability to target T cells, the main cells of the immune system [17]. However, the potential risks cannot be ignored: although gene integration tends to select safe regions, there is still the possibility of accidental destruction of key genes, resulting in carcinogenesis. Furthermore, the production process requires a complex multiplasmid assembly system, contributing to high production costs. Moreover, certain viral proteins may trigger immune responses or cytokine storms [18–20]. To address these challenges, research strategies continue to emerge. For example, by deleting the enhancer sequence, the design of self-inactivating vectors has been proven to be effective in reducing the risk of abnormal activation of adjacent genes [21]. The use of CRISPR–CRISPR-associated protein 9 (Cas9) technology to guide the targeted integration of LV vectors into safe genomic harbor loci, such as adeno-associated virus integration site 1, is another strategy to reduce the risk of insertion mutations [22]. In recent years, the development of integrase-deficient vectors has provided a solution that considerably reduces the risk of genomic integration [23]. Although LV vectors are associated with a low cancer risk in short-term treatment, safety assessment of their long-term application is very important. This requires close monitoring of the distribution of integration loci via high-throughput sequencing technology and the establishment of a complete long-term follow-up mechanism to assess long-term risk.

##### Retroviral vectors

Retroviruses (RVs) and LVs belong to the Retroviridae family, but RV vectors can infect only actively dividing cells. This type of vector transcribes the RNA genome into DNA via reverse transcriptase and integrates it into the genome of the host cell, thereby achieving long-term stable expression of exogenous genes [24]. Although RV vectors have shown great potential in early gene therapy, their application in in vivo CAR delivery still faces major challenges. The main problem lies in its strong random integration property, which can easily lead to the destruction of key genes. For example, in the famous X-linked severe combined immunodeficiency (X-SCID) treatment case in 2003, 2 children with X-SCID had a γ-RV vector inserted into the LIM domain only 2 (LMO2) proto-oncogene locus, which resulted in T-cell leukemia-like clonality [25]. In addition, RV vectors have the problems of a strong immune response and a low production efficiency [26]. In view of these safety issues, the current application of RV vectors is focused mainly on the preparation of CAR-T cells in vitro. If it is considered for in vivo delivery, it must be designed with strict safety switches and detailed risk assessments.

##### Emerging viral platforms

Scientists are genetically modifying viruses to overcome the limitations of traditional delivery systems, considerably improving their delivery performance. The key transformation strategies include capsid protein engineering, i.e., remodeling the viral coat to increase its organ-targeting ability; promoter optimization, i.e., achieving precise gene switching by customizing smart promoters; and immune evasion modification, e.g., by adding a polyethylene glycol “stealth coating” to avoid immune clearance [8,27]. These engineered viral platforms provide innovative solutions for the in vivo targeted delivery of CARs, but their long-term biological safety and scalability for large-scale production processes still need further verification.

#### Nonviral vectors

##### Lipid nanoparticles

As currently the most promising nonviral delivery vector, lipid nanoparticles (LNPs) automatically encapsulate nucleic acids via positively charged lipid molecules to form stable nanocomposites and can efficiently deliver various therapeutic molecules [28]. Their core advantages are reflected in multiple dimensions: they can increase the absorption rate of insoluble drugs by 3 to 5 times; the unique double-layer structure can effectively withstand light, oxygen, and enzymatic degradation; and their stability can be maintained for more than half a year at 4 °C. Crucially, by installing navigation devices such as the CD3 antibody on the surface, precise cell delivery can be achieved, and the risk of off-target effects can be greatly reduced [29]. In addition, their versatility supports the simultaneous encapsulation of messenger RNAs (mRNAs), small interfering RNAs, and small-molecule drugs, creating an ideal platform for combination therapy [30]. However, the clinical application of LNPs still faces important challenges. The drug-loading capacity is relatively low, making it difficult to meet the requirements for high-dose treatment [29]. In terms of stability, they are very sensitive to storage conditions. The pH should be strictly maintained at 7.4 ± 0.2, and the environment should be lightproof at 2 to 8 °C; otherwise, particle size growth and drug leakage can be easily induced. The safety assessment revealed that some cationic lipids may cause a mild inflammatory response, and the long-term toxicity still needs systematic verification [31]. The production process relies on sophisticated microfluidic technology, which places strict requirements on GMP-compliant production [32]. Notably, the introduction of a novel ionizable lipid process drove a breakthrough improvement in performance [33].

##### Polymer nanocarriers

This type of carrier is similar to a programmable smart capsule, which carries genetic drugs through electrostatic adsorption. Positioning devices can be installed on the surface to enable the uptake rate by specific cells to reach more than 80%. The polymer shell can also resist gene enzyme degradation [34]. More importantly, the drug release rate can be precisely controlled by adjusting the molecular weight and monomer composition of the polymer. Nevertheless, there are still bottlenecks in the application of polymer nanocarriers: approximately 30% of the carriers cannot completely release the drug due to overstability, and another 20% of the carriers cause drug leakage due to premature degradation [35]. In the production process, complex processes must be relied upon to control particle size and drug loading, resulting in high single-batch costs. In terms of safety, materials such as polyethyleneimine may cause dose-dependent cytotoxicity and are prone to particle size growth and aggregation during storage [36]. Coupled with stringent GMP requirements and the 18- to 24-month approval cycle, the pace of clinical transformation is considerably delayed [35]. Future directions include the introduction of pH-sensitive bonds, the optimization of the lyophilization process, and the use of machine learning to drive the structural design of polymers to overcome existing limitations [37].

##### Exosomes

This type of vector secreted by the human body has natural biocompatibility, and the CD47 “do not eat me” signal carried on the surface helps prolong its survival in the body [38,39]. Its unique function is that it can be guided to specific organs through membrane proteins and has the ability to penetrate the blood–brain barrier. However, in clinical applications, this technology faces 3 challenges: low production efficiency and obstacles to large-scale production, poor drug-loading efficiency, and insufficient stability [39]. Exosomes derived from different sources present distinct risks: although exosomes from CAR-T cells naturally carry the CAR structure, there is a risk of immune cell attack caused by antigen shift [40]. Exosomes from mesenchymal stem cells have low immunogenicity, but care should be exercised with respect to their potential tumor-promoting properties [41]; although exosomes from dendritic cells have been clinically verified to be safe, their production costs are high [42]. To achieve clinical translation, safety issues such as long-term genomic integration risk and changes in engineering immunogenicity must be systematically addressed while simultaneously promoting the standardization of production processes and accurate in vivo tracking technology. In particular, in the field of in vivo CAR delivery, there is an urgent need to establish a sound quality control and toxicological evaluation system.

#### Implantable bioscaffolds

Optimization of surface coating technology can control the degradation time of scaffolds, ranging from weeks to months [43]. Despite the important advantages of this technology, it still faces many challenges in clinical application: the implantation process requires surgical intervention, which not only increases the risk of infection but also prolongs the recovery cycle of patients; the scaffold material may trigger immune rejection, resulting in the formation of scar tissue, which impairs the function of CAR-T cells; in addition, the physical size and material properties of the scaffold limit the drug-loading capacity, making it difficult to achieve the sustained release of high doses of cells [43]. However, mouse model studies have shown that MASTER scaffolds can rapidly generate and release functional CAR-T cells, and their persistence in the body and antitumor effects exceed those of traditional therapies [2]. Currently, more clinical trials are urgently needed to verify their long-term safety and potential immune response to fully elucidate their true potential in the field of CAR-T-cell therapy.

In summary, the selection of delivery vectors is crucial for the success of CAR-T-cell therapy, with each type of vector having its unique advantages and challenges to overcome. Among viral vectors, LVs have become the mainstream for ex vivo preparation due to their large capacity of 8 kb and natural T-cell targeting, but their risk of genome integration must be strictly controlled through self-inactivating designs and CRISPR-guided safe harbor integration strategies. AAVs, while capable of achieving long-term expression and strong organ penetration, are limited by their capacity constraints, preexisting neutralizing antibodies, and the risk of liver toxicity at high doses. RVs have essentially been phased out of in in vivo delivery due to the important safety defect of random integration leading to leukemia. In the realm of nonviral vectors, LNPs have emerged as a new choice for in vivo applications due to their nonintegrating characteristics, the ability to modify CD3 antibodies for enhanced targeting, and the capacity to co-deliver multiple molecules, but their low drug-loading capacity and stringent storage conditions limit their application. Polymer nanocarriers can program drug release but face challenges with instability in drug release and material toxicity. Exosomes, with their natural biocompatibility and ability to cross the blood–brain barrier, have attracted attention, but their low drug-loading efficiency and difficulties in scaling up production hinder their clinical translation. Implantable bioscaffolds, by locally generating CAR-T cells in situ, avoid systemic toxicity and exhibit superior persistence in mouse models, but the risks of surgical implantation, material rejection, and physical limitations on drug loading require further breakthroughs. From the perspective of clinical translation, different vector systems show distinct differences: LVs are primarily used for the preparation of CAR-T cells ex vivo, LNPs are leading the innovation in in vivo mRNA delivery, while AAVs and scaffold technologies are still working to address safety and production process challenges [44].

## Disease Application: Opportunities and Preliminary Validation

### Hematological malignancies

#### B-cell malignancies (acute leukemia, chronic lymphoproliferative disorders, and multiple myeloma)

**Fig. 3.**
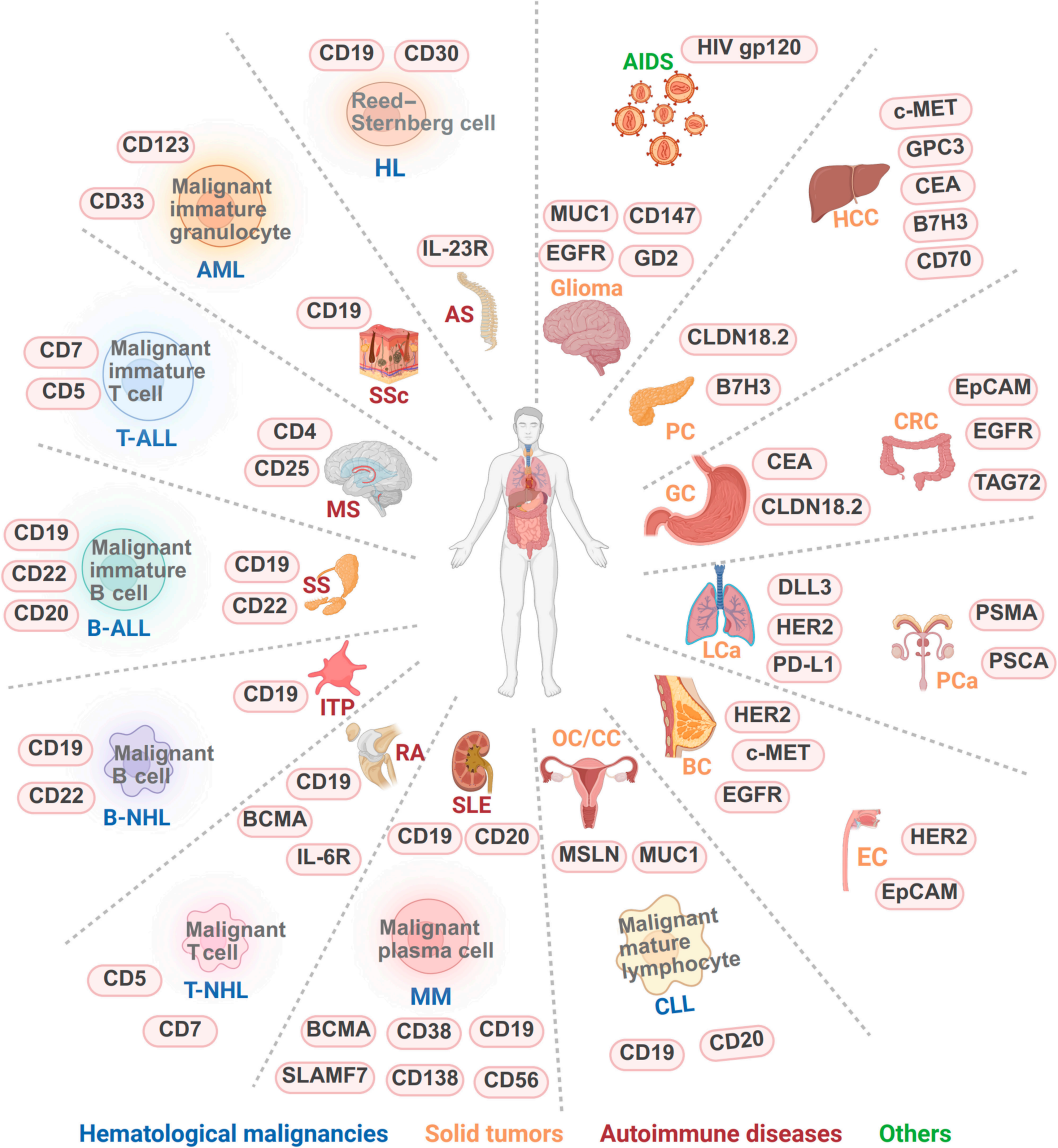
CAR-T therapy in diseases and related targets. B-ALL, B-acute lymphoblastic leukemia; T-ALL, T-acute lymphoblastic leukemia; AML, acute myeloid leukemia; MM, multiple myeloma; B-NHL, B-cell non-Hodgkin’s lymphoma; T-NHL, T-cell non-Hodgkin’s lymphoma; HL, Hodgkin’s lymphoma; BC, breast cancer; OC, ovarian cancer; CC, cervical cancer; CRC, colorectal cancer; PC, pancreatic cancer; GC, gastric cancer; HCC, hepatocellular carcinoma; EC, esophageal cancer; LCa, lung cancer; PCa, prostate cancer; AS, ankylosing spondylitis; SSc, systemic sclerosis; MS, multiple sclerosis; SS, Sjögren’s syndrome; ITP, idiopathic thrombocytopenic purpura; RA, rheumatoid arthritis; SLE, systemic lupus erythematosus; AIDS, acquired immunodeficiency syndrome; BCMA, B-cell maturation antigen; SLAMF7, signaling lymphocytic activation molecule F7; DLL3, delta-like ligand 3; EGFR, epidermal growth factor receptor; GD2, ganglioside 2; MUC1, mucin 1; HER2, human epidermal growth factor receptor 2; EpCAM, epithelial cell adhesion molecule; PD-L1, programmed death-ligand 1; MSLN, mesothelin; CEA, carcinoembryonic antigen; PSMA, prostate-specific membrane antigen; PSCA, prostate stem cell antigen; CLDN, claudin; GPC3, glypican-3.

Currently, in vitro CAR-T-cell therapy has achieved important efficacy in acute lymphocytic leukemia, non-Hodgkin’s lymphoma, and multiple myeloma, with an overall response rate of 82% to 100%, a complete remission rate that often exceeds 70%, and controllable safety (Fig. 3). The CRSs are mostly grades 1 and 2, and the incidence of immune-effector-cell-associated neurotoxic syndrome is low [45–49]. However, the problem of recurrence still exists, which has prompted researchers to turn to the development of in vivo CAR-T-cell therapy. This technology achieves repeated drug administration through transient expression, such as with mRNA nanoparticles, which has theoretical advantages in maintaining tumor control and reducing toxicity [50]. In preclinical research, a number of breakthroughs have confirmed the feasibility of in vivo-generated CAR-T cells for the treatment of B-cell malignant tumors. Researchers have successfully engineered a variety of immune cells with targeted LV vectors. Among them, vectors targeting CD8^+^ T cells can simultaneously produce 3 anticancer fighters in the body, CAR-T, CAR natural killers, and CAR natural killer T cells, and can completely eliminate CD19^+^ tumor cells in a xenograft tumor model [51,52]. Interestingly, CAR-T cells produced by targeting CD4 cells perform better in high-burden tumors, overcoming the limitations of traditional CD8^+^ T cells, which are prone to exhaustion [53]. In addition, the novel CD3-targeting vector can modify T cells without pretreatment, and the innovative platform UB-VV100 can regulate CAR-T-cell production on demand [54,55]. More advanced delivery systems, such as double-antibody-guided viral vectors, have continuously depleted B cells in monkeys for more than 10 weeks, showing great potential [56,57]. These studies have led to several important discoveries: first, multiple types of immune cells can be engineered simultaneously, broadening the range of treatment; second, CD4^+^ CAR-T cells exhibit a unique antitumor advantage, challenging the traditional treatment paradigm centered on CD8^+^ cells. A CRS similar to the clinical one was observed, suggesting that safety needs strict monitoring [52]. Optimized platforms such as VivoVec considerably enhance antitumor function by integrating costimulatory signals [57]. Currently, in vivo CAR-T therapy has entered the early clinical research phase. On July 2, Xu et al.’s team reported the first human data on the LV in vivo CAR-T pipeline ESO-T01 for the treatment of 4 patients with relapsed or refractory multiple myeloma in *The Lancet* (NCT0669685). The clinical results indicate that ESO-T01 has good safety, with no observation of grade 4 or higher CRS, and one patient experienced grade 1 immune-effector-cell-associated neurotoxicity syndrome. All 4 patients achieved an objective response rate of 100%, with 2 patients achieving stringent complete response and 2 patients achieving partial response. This marks an important milestone in the transition of in vivo CAR-T therapy from concept to clinical application [58]. Additionally, there are 2 ongoing clinical trials (NCT06528301 and NCT06539338) (Table 2). Although core issues such as vector specificity, toxicity control, and long-term activity still need to be resolved in this field, preclinical data have fully validated its potential as the next generation of CAR-T-cell therapy, especially for relapsed and refractory cases that require repeated dosing. Future breakthroughs will rely mainly on the continuous optimization of the targeted delivery system and the full validation of clinical safety.

**Table 2.** Ongoing in vivo CAR-T clinical trials

Disease	Clinical trial number	CAR target	CAR-T product name	Delivery system	Phase and estimated enrollment	Study duration
B-cell cancers	NCT06528301	CD19	UB-VV111	Lentivirus	I/106	2025–2029
B-cell cancers	NCT06539338	CD20	INT2104	Lentivirus	I/30	2024–2028
Autoimmune diseases	NCT06917742	CD19	CPTX2309	LNPs	I/38	2025–2026
Multiple myeloma	NCT06691685	BCMA	ESO-T01	Lentivirus	I/24	2025–2027
B-cell cancers	NCT07002112	CD19/CD20	LVIVO-TaVec100	Lentivirus	I/30	2025–2027

#### T-cell malignant tumors

In the field of T-cell malignant tumor treatment, the development of CAR-T-cell therapy presents unique challenges and opportunities (Fig. 3). Compared with the important efficacy achieved in B-cell malignant tumors, the application of in vitro CAR-T-cell therapy in T-cell tumors faces many bottlenecks: first, the source of autologous T cells is limited, and there is a risk of contamination by malignant cells; second, the in vitro expansion efficiency and low cost lead to high product heterogeneity. In addition, the long 3- to 4-week preparation cycle delays the timing of treatment. Currently, only in vitro CAR-T-cell therapies targeting limited targets, such as CD7 (NCT04689659) and CD5 (NCT03081910), have entered clinical trials, and the complete remission rate is generally less than 50%, which is far lower than the treatment effect on B-cell malignancies. This treatment dilemma has prompted researchers to turn their attention to in vivo CAR-T-cell technology, which has 3 advantages: first, it avoids the complicated processes of in vitro culture by directly transducing T cells from a patient in vivo; second, it can implement repeated dosing to maintain the therapeutic effect; and, third, the preparation cost and time are considerably reduced. More importantly, the transient expression characteristics considerably reduce the risk of long-term toxicity. A number of pioneering studies have provided strong evidence for this: a single injection of AAV-mediated in vivo CAR gene therapy delivered via AAV vectors can induce tumor regression in a humanized leukemia model, which not only simplifies the treatment process but also retains the diversity of normal T cells [59]. Owing to its excellent stability, DNA nanoparticle technology achieves “ready-to-use” gene delivery and provides convenience for clinical translation [60]. Of particular concern is the CD8-targeted LV system developed for angioimmunoblastic T-cell lymphoma, which successfully circumvents the cannibalism effect through accurate cell subgroup targeting and sheds light on the targeting of shared antigens such as CD4/CD5/CD7 [61]. To address the unique cannibalistic problem of T-cell malignant tumors, current research has focused on breakthroughs in 3 directions. In terms of delivery systems, the development of precise regulation technologies, including targeting nanoparticles, tissue-specific viral vectors, and transient mRNA expression, has improved target selection [62,63], and novel targeting strategies, such as T-cell receptor beta constant region 1/2 (TRBC1/2)-specific CARs (NCT03590574), have shown good potential; in terms of microenvironment modification, the co-expression of cytokines, such as interleukin-7/interleukin-15 (IL-15), effectively enhances the persistence of CAR-T cells [64]. With the continuous maturity of these key technologies, in vivo CAR-T-cell therapy is expected to become the first-line treatment option for T-cell malignancies, especially for patients who have failed traditional treatments or are in urgent need of rapid treatment.

### Solid tumors

CAR-T-cell therapy has been applied to the treatment of various solid tumors, including gastric cancer [65], melanoma [66], renal cell carcinoma [67], and pontine glioma [68] (Fig. 3). However, the objective response rate for ipilimumab in treating advanced melanoma is only 10% to 15%, indicating that the majority of patients exhibit primary resistance. Moreover, up to 70% to 80% of patients experience immune-related adverse events, including skin toxicities such as rashes in 40% to 50% of cases, gastrointestinal toxicities like diarrhea and colitis in 30% to 40%, hepatotoxicity in 10% to 15%, and thyroid dysfunction in 15% to 20%. Although the incidence of pneumonia is less than 10%, it can progress to fatal pulmonary damage [69]. The objective response rate for nivolumab in treating advanced non-small cell lung cancer is approximately 20% to 25%, with a toxicity profile characterized by skin reactions in 30% to 35%, gastrointestinal symptoms in 15% to 20%, and liver enzyme abnormalities in 10% to 15%. Notably, the incidence of pneumonia is as high as 5% to 10%, with a considerably increased risk in patients with underlying lung diseases [70]. The efficacy of traditional CAR-T-cell therapy in treating solid tumors is limited, mainly due to the inhibitory effect of the tumor microenvironment (TME) and insufficient infiltration of CAR-T cells. This challenge is currently being addressed by innovative in vivo CAR-T-cell technology. Through accurate local delivery, this technology considerably increases the degree of enrichment of CAR-T cells at tumor locations and greatly reduces systemic toxicity. Moreover, when combined with advanced biomaterials or nanocarriers, in vivo CAR-T-cell technology can directly remodel the immunosuppressive TME, creating a more suitable combat environment for CAR-T cells by releasing immune regulatory factors or degrading a dense extracellular matrix [63,71]. This regional delivery strategy enables CAR-T cells to adapt better to the tumor environment and overcomes the physical and immune barriers faced with systemic drug delivery. Recent breakthrough studies have provided important examples for in vivo CAR-T-cell treatment of solid tumors. Researchers have developed a CD5-targeting LNP delivery system to transiently generate antifibrotic CAR-T cells in vivo through the modification of mRNAs. In a heart failure mouse model, this innovative method achieved efficient mRNA delivery, and the generated CAR-T cells were enriched in the spleen and considerably improved cardiac fibrosis and functional recovery [72]. This result not only confirmed the therapeutic potential of in vivo CAR-T cells in solid tissues but also provided new insights for the treatment of other fibrosis-associated solid tumors. Notably, the efficacy of CAR-T-cell therapy in solid tumors is a key indicator for measuring the maturity of in vivo CAR-T-cell therapy. Future development includes the development of more precise targeted delivery systems, the exploration of new strategies for TME-specific regulation, and the optimization of the kinetic characteristics of the transient expression system. With the gradual breakthrough of these technical bottlenecks, in vivo CAR-T cells are expected to completely change the treatment landscape of solid tumors, providing new hope for survival in advanced patients. This innovative therapy is rapidly moving from the laboratory to the clinic, and its development trajectories are worth waiting for.

### Autoimmune diseases

The therapeutic field is undergoing a paradigm shift from ex vivo preparation to in vivo generation of CAR-T-cell therapy. In diseases such as systemic lupus erythematosus [73], neuromyelitis optica [62], systemic sclerosis [74], myasthenia gravis [75], etc. [76,77], ex vivo CAR-T-cell therapy has achieved preliminary success (Fig. 3). However, in vivo CAR-T technology, with its unique advantages, is gradually becoming a better treatment option. Compared with traditional methods, in vivo CAR-T-cell technology considerably simplifies the production process, effectively retains the natural functional and phenotypic characteristics of T cells, and avoids functional damage that may occur during the in vitro expansion process. The LNP-based transient expression system enables the regulatable expression of CARs and the possibility of repeated dosing. This “regulation on demand” characteristic is beneficial for the treatment of autoimmune diseases that require fine immune regulation [78], which is especially suitable. The latest research progress has injected new vitality into the field. In rheumatoid arthritis models, the use of an mRNA-CAR targeting the urokinase-type plasminogen activator receptor‌ antigen has successfully relieved inflammatory symptoms [79]. Another breakthrough is the development of universal targeted LNP technology, which can specifically reprogram CD8^+^ T cells in vivo and achieve “reset” of the immune system after B-cell depletion in a primate model. This discovery provides potential cures for various autoimmune diseases [80]. These innovative methods completely circumvent the lymphatic clearance preconditioning and complicated in vitro manufacturing processes required for traditional CAR-T-cell therapy, considerably improving the availability of treatment. With the initiation of the first clinical trial, CPTX2309, this technology officially entered the clinical translation stage. Future development will focus on 2 core directions: developing more accurate in vivo generation technologies for regulatory CAR-T cells and optimizing the transient expression system to achieve more controllable immune regulation. With the continuous innovation of delivery technology and the continuous accumulation of clinical data, in vivo CAR-T cells are expected to completely change the treatment landscape of autoimmune diseases, providing patients with safer and more convenient treatment options. This technological breakthrough not only represents an innovation in treatment methods but also heralds a change in the treatment concept from traditional immunosuppression to precise immune reconstruction.

### Others

In the treatment of infectious diseases, CAR-T-cell therapy represents a new treatment path, especially because it has unique advantages in treating viral infections such as AIDS that are refractory to traditional treatments (Fig. 3). The core mechanism of this technology involves the use of genetically engineered T cells to recognize and eliminate HIV infection precisely. Infected target cells: The CAR structure specifically binds to viral proteins expressed on the surface of infected cells or infection-induced host cell markers, thereby activating the killing function of T cells to clear the viral reservoir [81]. Unlike traditional antiviral drugs that can inhibit only viral replication, CAR-T-cell therapy is expected to achieve the complete elimination of infected cells, providing hope for a functional cure for AIDS. However, traditional CAR-T-cell therapies have shown limited efficacy in treating HIV. For example, the M10 CAR-T-cell therapy from Fudan University can considerably reduce viral load and suppress viral rebound to some extent, but it still fails to achieve a functional cure, and the risk of viral recurrence remains for patients [82]. A multicenter retrospective analysis of clinical data has revealed that the key factors restricting efficacy include insufficient CAR-T-cell persistence, viral diversity, and immune suppression [83]. In this special application scenario, in vivo CAR-T-cell technology has highlighted important value. The direct generation of CAR-T cells in vivo not only greatly reduces the cost of treatment but also, more importantly, enables rapid immune reconstruction, which is critical for controlling the window of viral replication. Currently, novel delivery platforms based on LNPs and mRNAs are constantly being optimized, providing reliable technical support for safe and efficient in vivo preparation of CAR-T cells. However, this innovative therapy still faces many challenges. HIV latent reservoir cells have low antigen expression characteristics, which places extremely high requirements on the recognition sensitivity of CARs; the high mutation rate of the virus may cause immune evasion; and at the same time, careful assessment of potential off-target effects and vector-related immune responses needs to be carefully evaluated [84]. Notably, for AIDS patients with already compromised immune systems, special attention needs to be paid to the risk of insertion mutations that may be caused by gene editing. Despite these technical bottlenecks, with the increasing precision of targeting strategies and the continuous optimization of vector systems, in vivo CAR-T-cell therapy is expected to lead to breakthrough treatment options for refractory infectious diseases such as AIDS, making a major leap from long-term viral suppression to a true functional cure.

## Key Scientific Challenges and Innovative Solutions

### Upgrading the delivery system: Efficient targeting and vector safety

To ensure efficient and precise delivery of CAR-T cells, overcoming the limitations of efficacy due to the nonspecific distribution of traditional carriers and the efficiency bottlenecks imposed by physiological barriers, researchers have developed a multidimensional delivery system upgrade plan, guided by the core strategy of T-cell targeting. The targeting design, based on T-cell-surface-specific markers, forms the core foundation: by covalently linking targeting elements, such as anti-CD3 antibodies, with delivery carriers, specific recognition is mediated through receptor interactions. For example, LNPs modified with anti-CD3ε antibodies are injected intravenously and precisely target T cells through antibody–CD3 molecule binding, primarily achieving efficient delivery in the spleen. Experiments have shown that when 200 μg of antibody is conjugated per milligram of liposomes, CAR expression increases with dosage, ultimately eliminating 90% of target B cells, thus paving a new way for the treatment of autoimmune diseases [85]. The nucleic acid aptamer strategy, an emerging alternative, employs single-stranded DNA/RNA fragments that are selected to specifically bind to T-cell surface markers, thus replacing antibodies. Its advantages encompass a smaller molecular weight and lower immunogenicity; however, it necessitates chemical modifications to enhance nuclease resistance and address instability within the body. The cell homing characteristic guidance strategy innovatively utilizes the biological trait of T cells to migrate toward inflammatory sites: by designing carriers responsive to chemokines in the immune microenvironment, they are directed to concentrate at lesion sites along a chemical gradient. This strategy complements marker targeting, and its technical breakthroughs present 3 synergistic mechanisms: dual-function CAR-T technology employs PEGylated poly(lactide-*co*-glycolide) nanoparticles encapsulating the signal regulatory protein alpha (SGRPα) gene, introduced into T cells via electroporation. The engineered anti-EGFRvIII-SGRP CAR-T cells have demonstrated a synergistic mechanism in glioma models: the CAR component directly kills tumor cells, while the secreted SGRPα protein blocks the “do not eat me” signal of tumor cells. Immunofluorescence revealed a 6.3-fold increase in the phagocytosis efficiency of immune cells within tumors after treatment. In lymphoma treatment, this technology has increased the cure rate and considerably reduced the expression of the T-cell exhaustion marker T-cell immunoglobulin and mucin domain-containing protein 3‌ (TIM-3) [86]; intelligently designed pH-sensitive CD3-targeting lipid bodies co-deliver CAR mRNA and interleukin-6 (IL-6) small interfering RNA, precisely targeting T cells after injection, achieving triple optimization in a leukemia model: maintaining CAR expression for over 90 d, achieving a cancer cell clearance rate of 98%, and controlling key inflammatory cytokines at safe levels [87]; implantable bioscaffolds disrupt traditional treatment paradigms, with an alginate scaffold featuring a 300-μm pore network converting 41% of recruited T cells into CAR-T cells within 24 h, while a collagen scaffold increases the accumulation of CAR-T cells at the tumor site by 8-fold, extending the survival of pancreatic cancer models from 28 to 67 d [88].

**Fig. 4.**
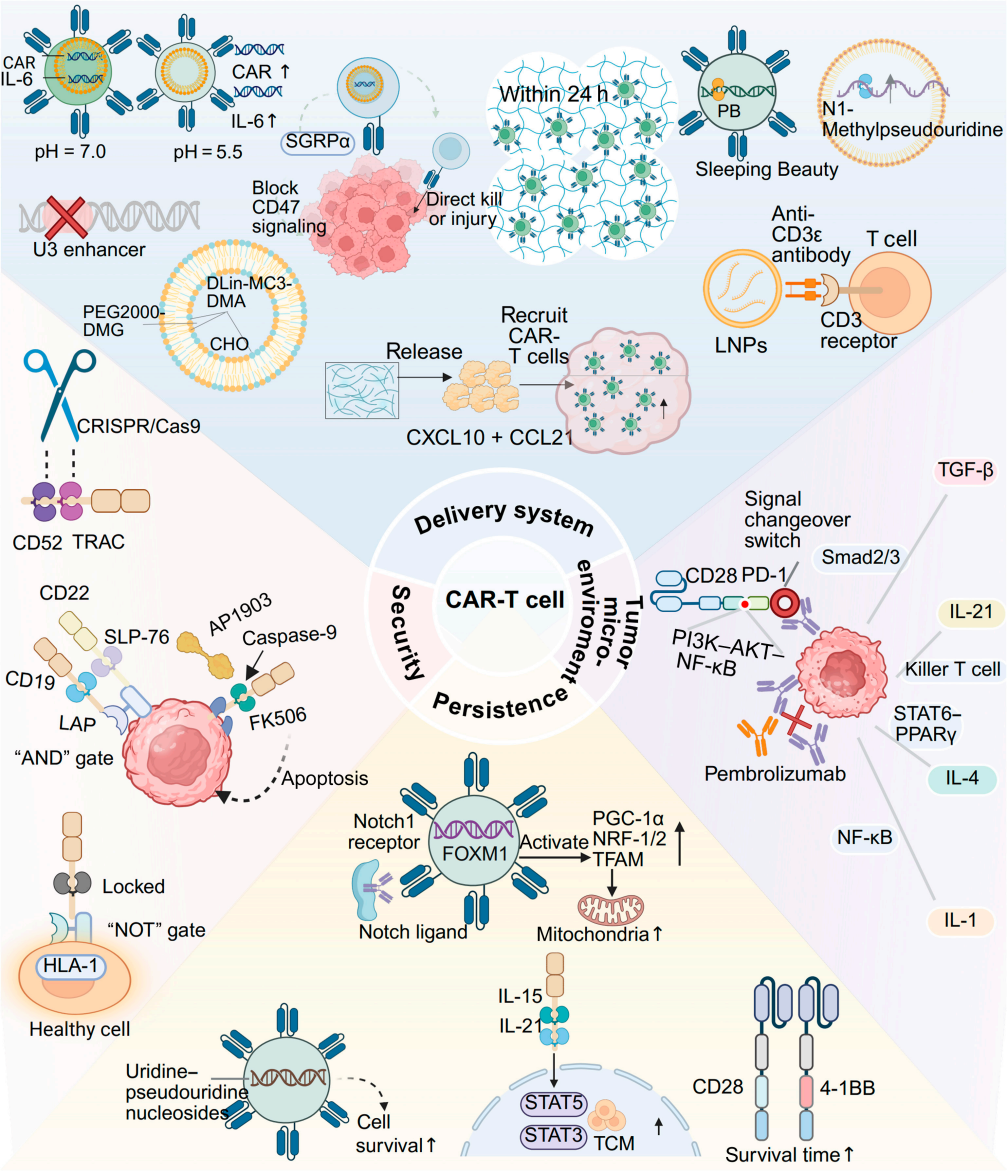
Key scientific challenges and innovative solutions in CAR-T-cell therapy. Optimization of delivery systems: antibody-coupled lipid nanoparticle technology to target T cells via the anti-CD3ε signaling module, bifunctional CAR-T to enhance the killing effect, simultaneous delivery of CAR messenger RNA (mRNA) and an interleukin-6 (IL-6) silencing element by a gene co-delivery system to enhance efficacy and control inflammatory response, alginate scaffolds to shorten the CAR-T-cell transformation time, collagen scaffolds to recruit and reprogram T cells in situ, viral vector deletion of the U3 enhancer through self-deactivation to reduce oncogenicity, optimized formulation of DLin-MC3 LNPs to reduce hepatic accumulation, transient expression of mRNA-N1-MeΨ, and the Sleeping Beauty transposon system, which utilizes the PB transposase gene to precisely implant CAR genes into genome-safe regions of the patient’s body. Strategies to enhance safety include utilizing a “with gate” linker for activation of T cells‌ (LAT), dual-targeted co-activation of SH2 domain-containing leukocyte protein of 76 kDa (SLP-76), implementing Tmod “nongate” autolocking to shield healthy tissues, employing an inducible caspase-9‌ (iCasp9) suicide switch for timely control of the storm factor, and preventing graft-versus-host disease‌ (GVHD) through CRISPR double knockout. Strategies to enhance persistence involve 4-1BB-CAR, which has a longer lifetime than CD28-CAR; GPC3-CAR, which co-secretes interleukin-15 (IL-15)/interleukin-21 (IL-21) and increases the central memory T cell‌ (TCM) ratio via the signal transducer and activator of transcription 5‌/3 (STAT5/3) axis; NOTCH–forkhead box M1‌ (FOXM1) metabolic reprogramming, which enhances cellular self-renewal by increasing the number of mitochondria; and the use of uridine–pseudouridine-modified telomerase mRNA to increase cell survival. Strategies to overcome the tumor microenvironment include the programmed cell death protein 1‌ (PD-1)/CD28 chimeric receptor, which converts PD-L1 inhibition into CD28 activation signaling to enhance efficacy; interleukin-1 (IL-1), which inhibits nuclear factor-κB (NF-κB) to enhance immune cell killing; interleukin-4 (IL-4), which promotes apoptosis via ‌signal transducer and activator of transcription 6‌ (STAT6)–peroxisome proliferator activated receptor gamma‌ (PPARγ); IL-21, which promotes T-cell killing and inhibits regulatory T cells (Tregs) to increase CAR-T-cell infiltration; and transforming growth factor-β (TGF-β) local end-mothers against decapentaplegic homolog 2/3 (Smad2/3), which decreases T-cell depletion. TRAC, T-cell receptor alpha constant; LAP, latency-associated peptide; CXCL10, C-X-C motif chemokine ligand 10; CCL21, C-C motif chemokine ligand 21; PGC-1α, peroxisome proliferator-activated receptor gamma coactivator 1-alpha; NRF-1/2, nuclear respiratory factor 1/2; TFAM, mitochondrial transcription factor A; PI3K, phosphatidylinositol 3-kinase.

In the realm of vector safety, technological innovation plays a crucial role. Self-inactivation technology has been applied to viral vectors, such as by deleting the U3 enhancer fragment in RVs, which considerably reduces the oncogenic risk. This modification decreases the likelihood of activating the proto-oncogene LMO2 by 40-fold. Large-scale clinical follow-up studies have confirmed that the incidence of vector-insertion-related mutant leukemia is controlled at less than 0.1% [8,89]. In the field of nonviral vector technology, the new generation of LNPs has achieved important performance improvements through optimized formulations. The ionizable lipid DLin-MC3-DMA is mixed with cholesterol at a 50:38.5 ratio, and 5% PEG2000-DMG is added to reduce immunogenicity. This design considerably reduces liver accumulation by 60% and extends circulation time to 8 h [90]. Currently, alternative vector systems are showing a trend toward diversification. The mRNA/LNP platform employs N1-methylpseudouridine modification to achieve transient expression within 72 h, completely avoiding the risk of gene integration. The Sleeping Beauty transposon system uses PiggyBac (PB) transposase to precisely insert the CAR gene into a safe genomic region. In the treatment of leukemia that relapsed after transplantation, this technology achieved a 78% complete remission rate without observing abnormal clonal expansion (Fig. 4) [91].

Looking ahead, the intelligent upgrading of delivery systems will focus on optimizing surface functionalization and in vivo pathways and constructing smart systems that dynamically respond to the microenvironment. The deep integration of nanotechnology and artificial intelligence (AI) will drive this process. Future vectors, integrating bio-responsive materials, immune-modulatory molecules, and miniaturized biosensors, will sense local changes and adjust targeting, release kinetics, and immune-evading properties in real time. This will enhance delivery efficiency and safety, ushering in a new era for CAR-T therapy.

### Innovations in safety mechanisms: Precise control and risk mitigation

In the field of CAR-T-cell therapy, ensuring the safety of treatment is very important. To address the core issue of off-target effects, logic gating technology is currently used, with the dual-verification mechanism considerably enhancing targeting. For example, the LINK CAR platform uses an “AND gate” design: the antibody fragments that recognize CD19 and CD22 are linked to the linker for activation of T cells‌ (LAT) and SLP-76 signaling proteins inside T cells, respectively. Only when cancer cells express these 2 targets at the same time can the 2 signaling pathways synergistically activate the killing program. In the lymphoma model, this design reduced the fratricide rate from 35% to less than 7%, considerably improving the safety of treatment [92]. As a complementary strategy, Tmod technology employs a “nongate” design: through the precise regulation of the 42 amino acid hinge region, the CAR is automatically locked when it encounters the healthy cell marker human leukocyte antigen class I (HLA-I). Animal experiments confirmed that this design effectively protects vital organs such as the liver [93]. In addition to the above-described preventive designs, important progress has also been made in emergency safety control systems. In the use of fibroblast growth factor receptor 4‌-CAR-T cells for the treatment of child rhabdomyosarcoma, the inducible suicide switch inducible caspase-9‌ was integrated: the sensing module was constructed by fusing the FK506-binding protein with the apoptosis execution protein caspase-9. Specifically, when the small-molecule drug AP1903 is injected, drug-induced protein aggregation triggers the apoptosis program. Key data have shown that more than 95% of CAR-T cells can be removed within 2 h after drug administration, allowing timely control of cytokine storms [94]. Furthermore, gene editing technology eliminates the risk at the source: in CTA101 uCAR-T cell injection‌ therapy, the T-cell receptor alpha constant gene and the CD52 gene of T cells are synchronously knocked out through CRISPR/Cas9 gene scissors to prevent graft-versus-host disease and to resist the effects of myeloablative drugs. In a clinical trial of relapsed leukemia, this technology reduced treatment-related mortality to 3.8%, and the complete remission rate reached 92% [95]. In the future, we will develop a novel in vivo imaging technology to monitor the distribution, activity, and persistence of CAR-T cells in real time and construct an intelligent safety control system that integrates multiple gating mechanisms and an adjustable suicide switch to achieve precise regulation of the therapeutic effect. These innovations will promote the development of CAR-T-cell therapy in a safer and more controllable direction, laying the foundation for the expansion of its clinical application (Fig. 4).

In the foreseeable future, the safety control mechanisms of CAR-T cells will continue to evolve toward greater intelligence and integration. On the one hand, the development of novel in vivo imaging and multimodal sensing technologies will enable precise, real-time dynamic monitoring of the distribution, activity, and persistence of CAR-T cells in the body, providing immediate data support for safety assessment. On the other hand, efforts will be made to construct intelligent safety control systems. By deeply integrating multiple logic gate mechanisms and tunable suicide switches, these systems will enable precise programming and dynamic control of the intensity and spatiotemporal distribution of therapeutic effects. These innovations will drive CAR-T therapy to establish a stronger and smarter safety net, considerably reducing risks and laying a solid safety foundation for expanding its clinical application to a broader population.

### Long-acting performance enhancement: Cell persistence and functional optimization

In the field of CAR-T-cell therapy, the key to achieving long-term efficacy is to prolong the survival of cells in the body. In terms of CAR molecular design, the selection of the costimulatory domain has a decisive effect on cell longevity. Studies have shown that when the intracellular domain of the CAR is integrated with the 4-1BB domain, the signaling balance is maintained by regulating A20 ubiquitinase, and the survival time of cells is considerably prolonged compared with that of CAR-T cells containing the CD28 domain, reaching 2.3 times greater. In the lymphoma model, after 180 d of infusion, the survival rate of 4-1BB-CAR-T cells still reached 15%, which was considerably better than that of CD28-CAR-T cells [96,97]. In addition, cytokine engineering strategies have also been shown to be effective at enhancing immune memory. In targeted therapy for hepatocellular carcinoma, glypican-3-CAR-T cells that co-express IL-15 and interleukin-21 (IL-21) activate the STAT5/STAT3 signaling pathway to stimulate central cells. The proportion of memory T cells (Tcm) increased to 68%. This type of cell continues to expand in the body for more than 120 d, which increases the survival rate of mice from 40% to 90% [98]. Furthermore, metabolic reprogramming technology endows cells with neonatal characteristics: by activating the NOTCH signaling pathway to induce the expression of the forkhead box M1‌ transcription factor, the mitochondrial production ability of CAR-T cells is increased by 2.8-fold. This modification enables cells to acquire self-renewal ability (Tscm phenotype) similar to that of stem cells, and in a leukemia model, the time to maintain a tumor clearance rate of more than 85% was extended for 4 weeks [99]. Notably, preparation process innovation is also critical to retain the youthful state of cells. The researchers used a new process to compress traditional 14-d culture to 72 h: first, CD3/CD28 magnetic beads were used to activate T cells at high intensity for 24 h, followed by switching to low doses of cytokines to avoid overstimulation; second, CD62L beads were used on the third day. The magnetic beads precisely selected the young cells. This “short-term low-stimulation” strategy completely retains the migration navigation ability and proliferation potential of cells. Animal experiments confirmed that after infusion, in vivo peak expansion was reached 7 d earlier, and the clearance efficiency of leukemia cells increased by 40% [100]. To fundamentally overcome the limit of cell lifespan, key progress has been made in antiaging engineering: the telomerase mRNA structure is stabilized by uridine–pseudouridine nucleoside replacement technology, and after being introduced into CAR-T cells by electroporation, the telomere protection structure at the end of the chromosome is 1.8-fold longer, and the expression of cellular senescence markers decreases by 67%. In a lymphoma model, modified cells survived for more than 100 d, and the tumor inhibitory effect increased by 55% (Fig. 4) [101].

In the days to come, research will focus on the editing of epigenetic regulatory factors to maintain the memory phenotype while analyzing the key molecular networks associated with persistence in combination with single-cell multiomics technology [102]. These innovations are expected to overcome the bottleneck of existing technologies and establish a new paradigm for long-acting CAR-T-cell treatment.

### Solid tumor defense decryption: Microenvironment modulation and synergistic intervention

The key to the treatment of solid tumors is to decipher the multiple defense mechanisms constituted by the TME [103]. In the field of immune checkpoint regulation, the dual-track strategy has achieved breakthroughs. On the one hand, the combination of programmed cell death protein 1‌ (PD-1) inhibitors such as pembrolizumab can release the brake signals of the immune system. On the other hand, researchers have innovatively designed the PD-1/CD28 chimeric switch receptor by precisely combining the extracellular recognition domain of PD-1 with the intracellular activation domain of CD28 through molecular splicing. When the vector carrying the receptor gene enters the CAR-T cells, genetic instructions are released by pH-sensitive lipids to overcome the endosomal barrier. These instructions are translated into proteins by ribosomes, catalyzed into the correct structure by endoplasmic reticulum folding enzymes, modified by glycans in the Golgi apparatus, and finally positioned on the cell membrane to expose the key signal-activating region (ITAM) of the CD3ζ chain. The functional receptor complex thus formed activates the phosphatidylinositol 3-kinase–AKT–nuclear factor-κB (NF-κB) signaling pathway through the YMNM structure of CD28, reversing the tumor’s inhibitory signal into an activation signal, driving the secretion of interleukin-2 and promoting T-cell proliferation [104]. Targeting the complex cytokine network, the multitarget synergistic intervention strategy offers important advantages: it inhibits the NF-κB signaling pathway by blocking interleukin-1, enhances the lethality of immune cells, and increases the efficiency of tumor cell lysis. A 40% increase in apoptosis can be induced in leukemia through the activation of interleukin-4, and animal models have shown a 78% reduction in tumor burden. The application of IL-21 promotes the proliferation of killer T cells, and simultaneously, the inhibition of regulatory T cells increases intratumoral CAR-T-cell infiltration by 5-fold in a colorectal cancer model; the neutralization of transforming growth factor-β blocks the mothers against decapentaplegic homolog 2/3 (Smad 2/3) phosphorylation pathway and effectively controls T-cell expression of the key depletion marker TIM-3 (Fig. 4) [105,106].

As we look ahead, the development of intelligent and responsive CAR-T cells that incorporate microenvironment-sensing elements and adaptive regulatory loops is essential. A multitarget joint intervention system should be constructed to simultaneously overcome physical barriers, metabolic inhibition, and immune escape. Additionally, single-cell spatial omics technology should be utilized to analyze the heterogeneity of the microenvironment, guiding the design of individualized treatment programs. These systemic innovations will propel CAR-T-cell therapy beyond the current bottleneck in treating solid tumors, paving new paths for overcoming various refractory tumors.

## Future Prospects

In the future, CAR technology will enter a new era of exponential growth. On the delivery side, the fourth-generation programmable LNPs, by virtue of their AI-driven modular architecture, triple the targeting efficiency with nanoscale precision, similar to a “biomissile”, and are the first to establish an organ-specific delivery paradigm in central nervous system tumors [107]. On the cell side, the universal CAR-T 2.0 uses CRISPR–CRISPR-associated protein 12a‌ (Cas12a) to knock out the HLA barrier at one time, and the photocontrolled switches and metabolic sensors are implanted to achieve programmable and remote-controlled “living drugs” [107]. At the same time, the treatment area is rapidly expanding to include solid tumors, autoimmune diseases, and chronic infections: CAR-M macrophages turn into “mini-tanks” to break through the barrier of fibrosis and provide cutting-edge weapons for solid tumors such as glioma; CAR natural killer cells rely on the induced-pluripotent-stem-cell-derived CD19/B-cell maturation antigen dual target QN-139b; the first clinical transformation in refractory diffuse cutaneous systemic sclerosis was completed, and its universal properties reduced the production cost by 80% [108]. At the industrialization level, the GMP 4.0 fully automated factory and the quantum computation-aided design platform have reduced the cost of a single treatment from US$500,000 to US$50,000, whereas CAR-T 3-dimensional bioprinting has realized the one-stop production of patient-specific preparations. With breakthroughs in epigenetic clock reset and mitochondrial rejuvenation technologies, “one infusion, lifetime cure” is expected to become a reality in the near future. This medical revolution, which is jointly driven by synthetic biology, nanotechnology, and AI, will eventually seal cancer and other chronic diseases in the history museum.
